# C-Reactive Protein Is Not Correlated With Endothelial Dysfunction in Overweight and Obese Women

**DOI:** 10.4021/jocmr1418w

**Published:** 2013-06-21

**Authors:** Raphael Ribeiro Sampaio, Ana Marice Ladeia, Romulo Bagano Meneses, Maria de Lourdes Lima, Armenio Costa Guimaraes

**Affiliations:** a Bahiana School of Medicine and Public Health, Bahia Foundation for the Development of Sciences, FBDC, Salvador, Bahia, Brazil

**Keywords:** Obesity, Endothelial dysfunction, Flow-mediated vasodilation, C-reactive protein

## Abstract

**Background:**

Obesity is a complex and multifactorial disease, has an inflammatory pattern and is associated with higher cardiometabolic risk. There are recent reports associating an elevated C-Reactive Protein (CRP) with a microscopic endothelial dysfunction. The objective is to evaluate if there is an association between serum levels of CRP and endothelial function in women with overweight/obesity, as well as the correlation between CRP and anthropometric variables.

**Methods:**

This is a cross-sectional study that analyzed secondary data from patients treated in an institution of tertiary education, as part of the weight excess and cardiometabolic disease survey. The study included patients with overweight/obesity who had CRP and endothelial function tests already made and inserted into the survey database. The endothelial function was evaluated by: reactive hyperemia test (endothelium-dependent vasodilation). All tests were recorded and later analyzed by the same echocardiographer who performed the examination. Statistical analyses were realized in the Statistical Package for the Social Sciences (SPSS) version 14. It was considered statistically significant a P value < 0.05.

**Results:**

This study included 47, nonsmoker women. with a BMI of 32.37 ± 5.06 kg/m^2^, median of CRP of 2.59 mg/L and flow-mediated dilation (FMD) of 8.75% ± 5.22%. There was no correlation between CRP and endothelial dysfunction in this population (*r_s_* = 0.08, P = 0.64). No correlation was observed between CRP and BMI. There were no differences of endothelial dysfunction variables and CRP in groups in use or not of medications (Hypolipidemic, antihypertensives and hypoglycemic agents).

**Conclusion:**

There was no association between CRP and FMD and this can suggest that it is possible that the level of eNOS dysfunction associated with increased CRP is not enough to lead to macroscopic changes and harm vasodilation.

## Introduction

Obesity inflammatory state has been a subject of great interest due its importance in the development of various associated pathological conditions, such as endothelial dysfunction [1-12].

This parainflammation and the oxidative stress produced by an excess of adipose tissue can lead to endothelial dysfunction by different ways [6, 10-14]. One of them would be by the increase of plasma C reactive-protein (CRP) - an adipokine and hepatic acute phase protein. There are evidences suggesting that this protein - historically considered merely an inflammatory marker - participates in the endothelial injury process by decreasing the bioavaibility of nitric oxide through an inhibition of nitric oxide sintase (eNOS) [8, 10, 12-15].

This study tested whether plasma C-reactive protein levels are associated with impaired endothelial function, measured by brachial artery flow-mediated dilation (FMD). Secondarily, we tested whether CRP levels are associated with anthropometric variables.

## Methods

This cross-sectional study included women with overweight or obesity treated in an ambulatory of obesity by a multidisciplinary team. These patients had CRP levels measured and endothelial function evaluated already made and entered into the database of a “clinical and epidemiological aspects of obesity” survey, in progress. Patients with CRP ≥ 10 mg/L, in use of anti-inflammatory drugs, or with clinical evidence of acute inflammation were excluded of this study.

After signing the informed consent form, patients were evaluated by interview and physical examination that included measures of body mass index (BMI), waist circumference (WC) and blood pressure (BP).

CRP dosage was performed preceding endothelial function test, through Nephelometry method in a clinical laboratory with technique quality certification established.

Endothelial function assessment was performed by an echocardiographist physician by high-resolution ultrasound. After 30 minutes rest, the subjects had been lying in supine position for 10 min in a stable-temperature room and then was performed a baseline measurement of brachial artery diameter in a longitudinal section (2 - 15 cm above the elbow). Reactive hyperemia was induced by occluding arterial blood by using a sphygmomanometer insufflated to 50 mmHg above the systolic pressure. After 5 min, the cuff was released. The arterial flow velocity was measured by a pulsed Doppler signal at a 60-degree angle to the vessel during the resting scan and for 15 s after the cuff deflated. The artery was scanned for 30 s before and 120 s after cuff release [16, 17].

Every patient had at least 4 hours of fasting before the exam. The mean and median of fasting time was 5.05 ± 1.4 and 4.5 hours, respectively.

All the exams was recorded and analyzed by the same ultrasonographer who performed the exam and who did not know the CRP levels of the patients.

The brachial artery diameter was measured from the anterior to posterior wall, with reference to the line between media and adventitia layers (“M line”). The mean diameter was calculated from measurements of three cardiac cycles coincident with the R wave on the electrocardiogram. Diameter changes were derived as percentage changes relative to the first scan. The blood flow was obtained multiplying the Doppler-pulsed blood velocity measurements by heart rate in the different stages of exam [17].

It was considered predictive variables of endothelial function: Flow-mediated dilation (FMD); mean flow velocity during reactive hiperemia (FMV); and absolute change in flow-mediated dilation (ΔFMD).

Statistical analysis was performed in the SPSS software version 14. Quantitative variables were expressed by mean ± standard deviation and by median and interquartile interval if the variables had a skewed distribution. Comparisons of two means or medians were performed by t-student test for independent samples and Wilcoxon-Mann-Whitney, depending of variable distribution profile. Correlations were performed by Pearson test for parametric variables and Spearman test for nonparametric variables. The statistical significance was defined by two-tailed test and P value < 0.05.

## Results

Among, 70 patients included in the survey, 47 women met the requirement of having CRP levels and endothelial function test in the database. Nine of them were excluded due CRP ≥ 10 mg/L. Table 1 shows clinical and demographic characteristics of 38 women with a mean age of 53 ± 11 years, predominantly non-white (86.8%), a mean BMI of 32.37 ± 5.06 kg/m^2^ and waist circumference of 104.8 ± 10.64. CRP median was 2.59 mg/L (interquartile range = 4.26 mg/L) and FMD mean of 8.75% ± 5.22% and FMV mean of 55.92 ± 24.84 cm/s.

**Table 1:** Clinical and Demographic Characteristics of Population (n = 38)

Age (years)	53.61 ± 11.61
Education Level^a^	
Illiterate, n (%)	1 (2.7%)
Complete First grade	16 (42.1%)
Complete Second grade	19 (50%)
Incomplete superior	1 (2.7%)
Skin Color^b^	
White, n (%)	4 (10.52%)
Non-White, n (%)	34 (86.8%)
Smoking^b^	
Non smoker, n (%)	36 (100%)
BMI^a^, kg/m^2^	32.37 ± 5.06
WC^b^, cm	104.833 ± 10.64
SBP^a^, mmHg	134.19 ± 17.435
DBP^a^, mmHg	86.16 ± 10.69
MBP^a^, mmHg	102.2 ± 11.8
Medication use	
Antihipertensives^c^, n (%)	25 (71.4%)
Hipoglycemiants^d^, n (%)	13 (39.4%)
Lipid lowering agents^d^, n (%)	14 (42.4%)
CRP, mg/L, median/interquartile range	2.59/4.26
FMV, cm/s	55.92 ± 24.84
FMD (%)	8.75% ± 5.22%
ΔFMD	0.302 ± 0.174

N, n: Sample number; BMI: Body mass index; WC: Waist circumference; SBP: Systolic blood pressure; DBP: Diastolic blood pressure; ΔT: Time interval; FMV: Flow-mediated mean velocity in reactive hiperemia; FMD: Flow-mediated vasodilation; ΔFMD: Absolute difference between arterial basal diameter and arterial diameter after reactive hiperemia. ^a^N = 37; ^b^N = 36; ^c^N = 35; ^d^N = 33.

There was no correlation between CRP and FMD (*r_s_* = 0.08, P = 0.64). There was a trend for positive association between CRP and BMI (*r_s_* = 0.309, P = 0.06) and between CRP and WC (*r_s_* = 0.281, P = 0.09).

Dichotomizing groups by CRP median (2.59 mg/L), there was no statistically significant differences on FMD (P = 0.6), FMV (P = 0.2) and ΔFMD (P = 0.4), as seen in Table 2.

**Table 2:** Assessment of Endothelial Function, in Groups Dichotomized at the Median Value of CRP

	CRP ≥ 2.59	CRP < 2.59	P
FMV, cm/s	60 ± 24.6	51.01 ± 24.7	0.2
FMD	9.2 ± 4.9%	8.3 ± 5.6%	0.6
ΔFMD, mm	0.33 ± 0.16	0.28 ± 0.18	0.4

FMD: Flow-mediated vasodilation; ΔFMD: Absolute difference between arterial basal diameter and arterial diameter after reactive hiperemia. FMV: Flow-mediated mean-Speedity in reactive hyperemia.

Serum CRP levels, FMD, FMV and ΔFMD were not different among users or non-users of antihypertensives, lipid-lowering and hypoglycemic agents (Table 3 and 4).

**Table 3:** Comparison of Serum CRP Level on Individuals Who Use Drugs or Not

	Drugs yes CRP (mg/L)	Drugs not CRP (mg/L)	P
Antihypertensives	2.60	1.565	0.75
Lipid-lowering agents	2.42	3.34	0.17
Hypoglycemiants	3.28	2.61	0.19

**Table 4:** Comparison Between Endothelial Function Variables in Individuals Using Medications or Not

	Yes	No	P
Antihypertensives			
FMV, cm/s	54.88	55.55	0.95
FMD	8.84%	8.40	0.83
ΔFMD, mm	0.3	0.32	0.79
Lipid-lowering agents			
FMV, cm/s	51.97	54.58	0.76
FMD	9.77%	8.67%	0.57
ΔFMD, mm	0.34	0.30	0.65
Hypoglycemiants			
FMV, cm/s	55.65	52.06	0.68
FMD	10.31%	8.37%	0.32
ΔFMD, mm	0.33	0.31	0.77

FMD: Flow-mediated vasodilation; ΔFMD: Absolute difference between arterial basal diameter and arterial diameter after reactive hiperemia. FMV: Flow-mediated mean-Speedity in reactive hyperemia.

## Discussion

C-Reactive Protein is an important cardiovascular risk marker and several studies have attempted to demonstrate that this is an active molecule and relevance in the process of Endothelial Dysfunction.

The population of our study was exclusively female, nonsmoker, mostly obese with abdominal adiposity and on medications used to treat conditions of high cardiovascular risk (systemic arterial hypertension, dyslipidemia and diabetes). The CRP median was 2.59 mg/L, corresponding to a band of moderate cardiovascular risk. Considering the risk profile of this study population, one would expect higher levels of CRP, as happened in a study by Visser et al that included 8,678 women and demonstrated that obese subjects had CRP 6.21 times higher than those of normal weight [10]. In the other hand, in Spearman correlation analysis was found a trend of association between CRP and waist circumference (P = 0.06) and between CRP and BMI (P = 0.09), which could suggest the important role of global or localized obesity in the subclinical inflammatory response. Endothelial function of this population was not significantly impaired, since the average of FMD was greater than normal (> 6.3%) in patients with significant cardiovascular risk [18]. Moreover, the following aspects should be emphasized: these women had a better health care than most obese people since they were assisted by a multidisciplinary team in an institution of tertiary education.

This study also demonstrated that there was no correlation between CRP and endothelial dysfunction in overweight and obese women. It is somewhat surprising and disagrees with what has been suggested by recent literature. Hein et al demonstrated in animal model that CRP leads to uncoupling of endothelial nitric oxide sintase (eNOS) and promotes oxidative stress [13]. Two others authors, in different publications, demonstrated that in cellular models CRP leads to an inhibition of eNOS [14] and in the presence of aldosterone leads to a hyperactivation of epithelial sodium channel (eNAC) [15]. Endothelial nitric oxide sintase (eNOS) is the main vascular endothelial nitric oxide synthesizer, so it was expect that the decrease in its activity would be followed by an impaired vasodilator capacity and in the other hand a hyperactivation of eNAC lead to a vascular stiffening and thus ED. In the other hand, a study that included 1,154 male firefighters without known atherosclerotic disease, which sought to demonstrate whether there was an association between CRP and FMD, reached the same results of this present work - There was no relationship between these variables [19]. However it is important to emphasize that the results observed in animals and cell cultures cannot necessarily be extrapolated to human models.

Other endothelial function variables were tested in this study, such as mean speed flow-mediated (FMV) and none of them were associated with CRP. However, a study in nine men, with similar clinical characteristics of our study population, demonstrated a strong positive correlation between CRP and the decline of brachial blood flow during infusion of a inhibitor of eNOS (*r* = 0.85, P = 0.004) [20], suggesting an association between these variables. On the other hand, it seems precipitated to extrapolate the results from this study and conclude that subjects with increased CRP levels have more endothelial dysfunction. The author did not analyzed endothelial function in nature, but under the effect of an inhibitor of eNOS, so what could be concluded is that subjects with increased CRP levels have increased sensitivity to this inhibitor.

There was no difference of CRP between users or nonusers of lipid-lowering agents. The current literature suggests that statin use leads to a reduction in serum levels of CRP [21-24]. PRINCE trial, a intervention cohort, has observed a reduction of 16.9% of serum CRP levels after 24 weeks of pravastatin use compared with placebo (P < 0.001) [21]. That study was planned to analyze the effect of an intervention (pravastatin) over biological markers, differing of this cross-sectional study, in which is impossible ensure the regular medication use, as it is not know when it was started statin therapy and there is an elevated possibility of information bias.

This study also has not observed show differences of endothelial function variables between users or nonusers of antihypertensive, hypoglycemiant and lipid-lowering agents. It is an attractive field of research, and recent studies indicate that use of statins improves endothelial function by increasing the expression of eNOS and thereby increasing nitric oxide bioavailability [25]. RECIFE trial analyzed if a quickly cholesterol decrease after a ischemic event was associated with a better endothelial function and it was observed in the pravastatin group a increase com FMD of 42% after 6 weeks of treatment compared with placebo (P = 0.02) [26]. The divergence of this study with those cited above may have occurred because this one was a cross sectional study and do not controlled the regularity and duration use of the medication. In the other hand, the fact of there was no significant differences in endothelial function with or without medication (that could interfere with vascular function) suggests that the results are not biased by these variables.

Therefore, the present study showed no association between CRP levels and endothelial dysfunction assessed by flow-mediated dilation and a trend of association between CRP and BMI and WC. The lack of association between CRP and endothelial function in overweight and obese women brings new questions and hypotheses: 1), it is possible that the level of eNOS dysfunction associated with increased CRP is not enough to lead to macroscopic changes and compromise vasodilatation; 2), considering that the increase in CRP was moderate and that endothelial function was considered within normal limits, maybe they were really a population of obese with no impaired vascular function enough to change an imaging method of assessing subclinical atherosclerosis. Thus, this study open new perspective for studies evaluating similar population and that reproduce the same methodology analysis of endothelial function.
